# The Screening Strategy and Activity Investigation of Skipjack Tuna (*Katsuwonus pelamis*) Umami Peptides Based on Computer Simulation Prediction and Experimental Hydrolysis

**DOI:** 10.3390/foods14213777

**Published:** 2025-11-04

**Authors:** Qiufeng Song, Panpan Wang, Yue Li, Weiliang Guan, Luyun Cai

**Affiliations:** 1 Ningbo Global Innovation Center, Zhejiang University, Ningbo 315100, China; songqiufeng2021@163.com (Q.S.); 18867641480@163.com (P.W.); 2 Engineering Research Center of Bio-Process, Ministry of Education, School of Food and Biological Engineering, Hefei University of Technology, Hefei 230009, China; 3 College of Light Industry and Food Engineering, Guangxi University, Nanning 530004, China; 4 Hade College, Ocean University of China, Qingdao 266100, China; liyue@ouc.edu.cn

**Keywords:** computer-simulated hydrolysis, umami peptide, molecular docking, molecular dynamics simulation

## Abstract

Marine-derived proteins are important sources in the preparation of umami peptides due to their delicious and unique taste. The research endeavored to elucidate the established umami peptide library derived from Skipjack tuna protein through a combined approach of computational and experimental proteolysis. A total of five potential shared umami peptides (GVGGHGAGG, GVTGVG, GGVAGCQGK, MANR, and SPAAK) were identified through database and molecular docking, which revealed that hydrogen bonds and electrostatic forces critically influence the interaction between peptides and T1R1/T1R3. The specific amino acids within the T1R1/T1R3 corresponding to glutamic acid, serine, arginine, aspartic acid, and histidine significantly influenced the affinity for umami peptides. It was verified through sensory and electronic tongue analysis that all these peptides exhibit umami performance and flavor-enhancing effects. Furthermore, bioinformatic predictions and computer simulations exploring the biological activity of umami peptides revealed that GVGGHGAGG, GVTGVG, and GGVAGCQGK, combined with Keap1, presented potential antioxidant activity. These observations offered new insights for identifying bioactive umami peptides from aquatic products and a theoretical foundation for developing novel seasonings.

## 1. Introduction

Umami is recognized as one of the five fundamental flavors alongside sour, sweet, bitter, and salty [1,2]. Umami components extend beyond sodium glutamate and include other amino acids, peptides, and organic acids in protein-rich foods such as fish and shellfish. As a fundamental taste sensation, umami not only enhances our sensory experience of food but also plays a crucial role in seasoning and food processing [3]. Umami peptides promote the Maillard reaction, which enhances overall flavor perception compared to other umami-presenting substances. In addition, the high permeability and low bioavailability contribute to the strong flavor-enhancing ability with rapid absorption rates of the umami peptide. Umami peptides are small-molecule peptides derived from proteins or synthesized from amino acids [1]. Research indicated that the intricate relationship between umami peptides and their specific receptors, including the heterodimer T1R1/T1R3 receptor complex and metabolic glutamate receptors like mGluR1/mGluR4, can elicit the sensation of umami taste [4]. Umami-presenting substances like glutamate and 5′-ribonucleotides can bind to these receptors, triggering conformational changes within them, thus activating taste signal transduction pathways [5]. The T1R1/T1R3 receptor, as the main receptor for bright flavor perception, has a relatively high expression in the oral mucosa and can be more easily detected by choosing T1R1/T1R3 as the research object. In addition, the function of T1R1/T1R3 has been more fully studied compared with other receptors. Therefore, choosing T1R1/T1R3 as the research object can provide a better understanding of its mechanism of action on umami flavor perception.

The current methods for preparing umami peptides present limitations, involving multiple separation and purification stages that tend to be costly and time-consuming [6]. Recently, advanced computer technology has emerged as an alternative strategy for effectively screening umami peptides. Online tools such as BIOPEP-UWM [7] and Peptide Cutter [8] can simulate the enzymatic hydrolysis of specific proteins and predict their potential functional activity. Additionally, machine learning (ML) and deep learning (DL) methodologies, utilizing advanced prediction models for umami peptides, have been deployed on various online platforms, including iUmami-SCM [9], umami-mrnn [10], and UMPred-FR [11].

Skipjack tuna (*Katsuwonus pelamis*) is primarily processed into sashimi and canned fish meat as a valuable marine protein resource. During processing, a large number of byproducts of meat are generated, which are further hydrolyzed to yield bioactive polypeptides or small-molecule peptides [12]. The meat protein-derived peptides are commonly utilized in functional foods due to their potential for health benefits [13,14]. Xin et al. [15] employed computational virtual proteolysis and bioinformatics to screen novel antioxidant and anti-inflammatory peptides from bovine  hemoglobin, which was confirmed by cellular experiments to effectively mitigate LPS-induced oxidative stress and inflammation in RAW264.7 cells. In research by Yuxiang et al. [16], six novel umami peptides were identified in porcine type I collagen through virtual screening, sensory evaluation, and molecular docking simulations. Furthermore, collagen-rich squid skin-derived peptides demonstrated potential to activate the Keap1-Nrf2 pathway, exhibiting antioxidant activity by scavenging free radicals and promoting overexpression of cellular antioxidant enzymes [17]. Consequently, skipjack tuna protein may serve as a source of umami peptides with potential biological activity. Nonetheless, there has been a noticeable lack of studies focused on umami peptides obtained from Skipjack tuna, especially regarding the mechanisms behind their taste presentation. Using molecular docking, umami peptides that exhibit high binding affinity with umami receptors can be selected from numerous candidate substances [18]. Nevertheless, it should be noted that molecular docking may produce false positive results, thereby significantly restricting its application in screening for umami peptides [19]. To ensure the reliability of screening outcomes, the results obtained through molecular docking can be further validated using molecular dynamics (MD) simulation [20]. Combining molecular docking with molecular dynamics (MD) simulations provides a robust approach for scrutinizing the binding attachment and stability of umami peptides to T1R1/T1R3 receptor proteins that remain underexplored.

This study established a peptide library from skipjack tuna protein sources through a combination of computer simulation and experimental enzymatic hydrolysis, utilizing bioinformatics analysis alongside chromatography and mass spectrometry for separation and identification. Peptide sequences with potential umami properties were evaluated via analytical docking and molecular dynamics simulation.,Ttheir mechanisms of actionwere then analyze followed by validation of umami characteristics achieved through sensory evaluation and electronic tongue analysis. Eventually, molecular docking was employed to investigate the biological activity of umami peptides.

## 2. Materials and Methods

### 2.1. Material and Database

The Skipjack tuna was purchased from Ningbo Today Food Co., Ltd. (Ningbo Today Food Co., Ltd., Ningbo, China). After removal of skin, visible fat, and connective tissues, the cleaned muscle was minced, vacuum-packed, and stored at −80 °C until use. Samples were thawed at 4 °C, homogenized, and immediately subjected to enzymatic hydrolysis to minimize protein oxidation and degradation. The NCBI (https://www.ncbi.nlm.nih.gov/) and UniProtKB (https://www.uniprot.org/) databases were utilized for this study. The umami peptides with a purity of over 98% were synthesized by Sangon Biotech Co., Ltd. (Sangon Biotech Co., Ltd., Shanghai, China). The remaining chemicals and solvents used in the study qualified as analytical quality.

### 2.2. In Silico Hydrolysis of Skipjack Tuna

The sequence of amino acids for the *Katsuwonus pelamis* protein, which was identified as BBD78421.1, was retrieved from NCBI’s repository. *In silico* hydrolysis was conducted using the ExPASy PeptideCutter (https://web.expasy.org/peptide_cutter/, accessed on 24 February 2024) [8]. Enzyme-specific parameters were set according to the default full-specificity mode. Enzyme parameters were set as follows: pepsin (pH > 2.0, cleavage after Phe, Tyr, Trp), trypsin (Lys, Arg, except before Pro), and chymotrypsin A (Tyr, Trp, Phe, Leu, Met). Peptides with 3–15 residues were retained for further screening.

### 2.3. Prediction of Water Solubility and Toxicity

The peptide property calculator from the Innovagen web server (https://www.innovagen.com/proteomics-tools, accessed on 24 February 2024) was utilized to forecast the water solubility of chosen peptide sequences. An in silico technique called ToxinPred was created to forecast and create both hazardous and non-toxic peptides. The assessment of peptide toxicity was carried out in conjunction with ToxinPred (https://webs.iiitd.edu.in/raghava/toxinpred/, accessed on 25 February 2024) [21].

### 2.4. Umami Characteristic Screening

The main factors affecting the flavor profile of umami peptides are molecular weight, peptide length, and amino acid makeup. We preliminary screened the peptide sequences based on amino acid characteristics and then predicted the flavor characteristics of the peptides using six machine learning predictors based on combinatorial sequences, including UMPred-FRL (https://pmlabstack.pythonanywhere.com/UMPred-FRL, accessed on 26 February 2024) [11], Umami_YYDS (https://tastepeptides-meta.com/Umami_YYDS, accessed on 26 February 2024) [22], TastePeptidesDM (http://tastepeptides-meta.com/TPDM, accessed on 26 February 2024), BIOPEP-UWM (https://biochemia.uwm.edu.pl/, accessed on 26 February 2024) [7], Umami_SCM (https://camt.pythonanywhere.com/iUmami-SCM, accessed on 26 February 2024) [9], and Umami-MRNN (https://umami-mrnn.herokuapp.com/, accessed on 26 February 2024) [10].

### 2.5. Preparation and Separation Purification of Skipjack Tuna Protein Hydrolysate

Skipjack tuna protein hydrolysate (SPH) was prepared employing a two-step enzymatic hydrolysis method. It was obtained by collecting the meat of skipjack tuna, followed by washing, mincing, alkali degreasing, and grinding pretreatment. First, pepsin (30,000 U/mg) was added at an enzyme-to-substrate ratio of 3000 U/g, applied at pH 2.37 and 37 ° followed by heating the product to 100 °C for enzyme inactivation. The above-mentioned sample was cooled and then subjected to a second enzymatic hydrolysis step with trypsin (2500 U/mg) at a dosage of 2000 U/g under 37 °C for 2 h. Once the reaction was finishedn, the enzyme activity was terminated by high-temperature heating. The supernatant was collected by centrifugation to obtain crude protein hydrolysate, which was then freeze-dried for further analysis. The degree of hydrolysis (DH) was monitored by the OPA (o-phthaldialdehyde) method described by Fu et al. [23], and calculated based on the release of free amino groups. The average DH values of the obtained hydrolysate were determined to be 28.4 ± 0.8%.

The SPH lyophilized powder was reconstituted with ultrapure water and sequentially separated using ultrafiltration centrifuge tubes with 10 kDa and 3 kDa molecular weight cutoff. As a result, three molecular weight fractions were obtained as >10 kDa, 3–10 kDa, and <3 kDa fractions, which were freeze-dried for subsequent processing. The fraction exhibiting optimal flavor characteristics was subjected to fractionation via G-15 dextran gel chromatography, eluted with ultrapure water at a flow rate of 0.5 mL/min, with detection at 220 nm.

### 2.6. Sequence Identification by LC-MS/MS

Peptide sequence analysis was performed in accordance with previously established methods [24]. The purified peptide samples were redissolved in 0.1% formic acid aqueous solution and analyzed using nano-high-performance liquid chromatography-electrospray ionization tandem mass spectrometry (nanoHPLC-ESI-MS/MS).

### 2.7. Molecular Simulation

#### 2.7.1. Construction and Optimization of Protein Receptor

The ZDock server (https://zdock.umassmed.edu/) [25] was deployed to join T1R1 and T1R3 crystal models to produce a complete 3D protein structure. The T1R1 and T1R3 models were initially built using the protein structure prediction program AlphaFold2 (https://alphafold.ebi.ac.uk/) [26]. The constructed model of the TIR1/TIR3 receptor protein was not yet exemplary; therefore, it was optimized using 100 ns MD simulations after the protein model was established. The topology file containing the molecular dynamics systemwas generated from the prospective data. The simulation was performed utilizing the FF99SB-ILDN force field and the Tip3p water model as the solvent under a steady temperature of 300 K and a pressure of 1 bar. The total charge of the system was neutralized by introducing a suitable amount of Na^+^ ions, and energy minimization was achieved using the most aggressive descent approach. Subsequently, the system undergoes both the isothermal isovolumic ensemble (NVT) and the isothermal isobaric ensemble (NPT) equilibrium processes, each lasting for 100,000 steps. A 0.1 ps coupling constant was employed, with a time frame of 100 ps. Following this, a free molecular dynamics simulation (MD) was executed over 5000,000 steps, each lasting 2 fs, culminating in a total simulation time of 100 ns.

#### 2.7.2. Molecular Docking

The screened prospective umami peptides were initially converted to PDB format employing the Robetta server [27]. The Force Field (GAFF) and Steepest Descent methods were employed to optimize the structure of each peptide in Avogadro software (1.102.1) [28]. DiscoveryStudio 2019 client’s CDOCKER semi-flexible docking was utilized to molecularly dock each peptide to the T1R1/T1R3 receptor. The GetBox plugin 2018 was used to design the molecular docking box center coordinates (22.5, −8.4, −16.5), and the T1R1 cavity dimensions were 30 × 30 × 30. After docking, the ligand-receptor interactions were visualized and analyzed using PyMol software (2.1.0) and Discovery Studio 2019 [29].

#### 2.7.3. Molecular Dynamics (MD) Simulation

The Gromacs 2022.3 program was employed for the MD simulation. AmberTools22 was employed for small-molecule preprocessing to implement the GAFF force field, whereas Gaussian 16W handled the hydrogenation of small molecules and calculated the RESP potential. Energy minimization (steepest descent, 50,000 steps; force tolerance 1000 kJ·mol^−1^·nm^−1^) was followed by NVT (300 K, 100 ps, V-rescale thermostat) and NPT (1 bar, 100 ps, Parrinello-Rahman barostat) equilibration with positional restraints (1000 → 500 kJ·mol^−1^·nm^−2^). Production MD (100 ns, 300 K, 1 bar) employed LINCS constraints (2 fs timestep), PME electrostatics (1.0 nm cutoff), and van der Waals interactions (1.0 nm cutoff). Trajectories were analyzed for RMSD, RMSF, Rg, SASA, hydrogen bonds, and FEL using GROMACS 2022.3 built-in tools.

### 2.8. Peptide Synthesis

Peptides were synthesized using the standard Fmoc solid-phase method on a 2-chlorotrityl chloride (2-CTC) resin (0.8 mmol/g, GL Biochem, Shanghai, China). Each Fmoc-protected amino acid (4 equiv) was activated by HOBt/DIC (4 equiv each) in DMF and coupled for 1 h at room temperature. Fmoc groups were removed with 20% piperidine in DMF, and final cleavage was performed using a TFA/TIS/H_2_O (95:2.5:2.5, *v*/*v*/*v*) cocktail for 2 h. Crude peptides were precipitated with cold diethyl ether, lyophilized, and purified by reverse-phase HPLC (Waters 2695-2487 system, Agilent ZORBAX SB-C18 column, 4.6 × 250 mm × 5 µm, Agilent, Santa Clara, CA, USA) using a 5–95% acetonitrile gradient containing 0.1% TFA. Data acquisition and processing were performed using Waters MassLynx software (v4.1).

### 2.9. Sensory Evaluation

The polymer chain was then dissolved in ultrapure water to produce a concentration of 1 mg/mL sample solution, which was filtered using a 0.22 µm filter membrane. The resulting filtrate served as the evaluation sample solution. The sensory evaluation conducted by the method outlined in Zhuang, et al. [30] with some modifications. The sensory evaluation panel comprised ten trained members (five males and five females, aged 18–28) who had undergone formal sensory training following ISO 8586-2012 ((*Sensory analysis—General guidelines for the selection, training and monitoring of selected assessors and expert sensory assessors*) guidelines (International Organization for Standardization, Geneva, Switzerland, 2012).) (guidelines. All panelists had participated in at least two standardized training sessions to recognize and differentiate the five basic tastes (sweet, bitter, sour, salty, and umami) using graded reference solutions. Prior to the evaluation, each panelist demonstrated at least 80% accuracy in identifying reference samples and was familiar with intensity scoring on a 0–10 scale. Regular refresher training was provided to maintain assessment consistency. The sensory study at Zhejiang University was exempted from ethical approval, considering the minimal risk involved, while strictly adhering to *International* Ethical Guidelines for Health-related Research Involving Humans (Council for International Organizations of Medical Sciences, Geneva, Switzerland, 2016). This study obtained informed consent from all participants, and a standardized protocol was implemented throughout the experiment to ensure voluntary participation, anonymity, and the right to withdraw at any time. To assess several flavors, including sweet, bitter, sour, salty, and umami, reference substances such as sucrose (1%), quinine (0.25%), citric acid (0.08%), sodium chloride (0.35%), and monosodium glutamate (0.35%) were employed, respectively [31]. Additionally, the detection threshold of the peptide was determined via a triangular test by progressively diluting the peptide solution to 1 mg/mL [32]. The peptide’s umami-enhancing impact was evaluated as well. These peptides (0.4 mg/mL) were solubilized in 0.35% MSG solution, and their umami intensity was measured following established protocols, with results compared against the same concentration of MSG [16].

### 2.10. Electronic Tongue Measurement

Each peptide that was synthesized was solubilized in deionized water at a 0.1 mg/mL concentration. The quantification of these peptide solutions was carried out utilizing the sensor probes of the taste sense apparatus. A standard solution containing 0.3 mM tartaric acid and 30 mM KCl served as the reference solution. The sensor and electrode were engaged and positioned on the apparatus. Prior to each sample being tested, the sensor and electrode were rinsed with the cleaning solution for electrodes and then placed in the reference solution to monitor the membrane potential until it reached a stable level. To guarantee data stability, each sample underwent four tests; the initial test data were discarded, and the final three measurements were evaluated [33].

### 2.11. Prediction of Peptide Antioxidant Activity Based on BIOPEP-UWM and Molecular Docking Analysis

In reference to the method for screening bioactive compounds by Zhao et al. [31], the potential functional activities of peptides obtained through simulated hydrolysis were predicted using the “Bioactive Peptides” module in the BIOPEP-UWM online database (https://biochemia.uwm.edu.pl). With the aim of screening umami peptides exhibiting antioxidant activity, the crystal structure data of Keap1 were obtained from the RCSB Protein Data Bank (http://www.rcsb.org). Subsequently, molecular docking was performed between Keap1 and the target peptides.

### 2.12. Statistical Analysis

Results were derived from threeindependent experiments and presented as the average ± standard deviation. The statistical evaluation of the data was performed utilizing SPSS version 19.0 software, employing One-way Analysis of Variance (ANOVA) and Tukey’s test for significant differences at the *p* < 0.05 threshold.

## 3. Results and Discussion

### 3.1. In Silico Proteolysis of Skipjack Protein

ThesSkipjack protein exhibited a composition of 842 amino acids, and by simulating in silico hydrolysis, 114 peptide sequences were collected for the following analysis [33]. Umami peptides typically featured a primary amino acid chain of -O) (C)_n_ (O-, (n = 3–9) with an abundance of various hydrophobic amino acids and molecular weights less than 1 kDa [34,35]. These hydrophobic residues (e.g., Ala, Val, Leu, Gly, Pro) facilitate van der Waals and alkyl interactions within the hydrophobic pocket of the T1R1/T1R3 receptor, stabilizing the peptide-receptor complex and promoting Venus Flytrap Domain closure, thereby enhancing receptor activation and umami perception [36,37]. These interactions stabilize the peptide-receptor complex and contribute to binding affinity, as reported in previous structural studies of T1R1/T1R3 [31,38]. The criteria for typical umami peptides were met by the 75 peptide sequences that were checked for molecular weights, general formulas, and hydrophobic amino acids before moving on to further study. In addition, the in silico hydrolyzed peptides were also screened on account of their predicted basic properties, such as water solubility and safety (Table S1). The results demonstrated that all of the peptides were in demand for edible safety.

### 3.2. Virtual Screening of Potential Umami Peptides

**Figure 1:**
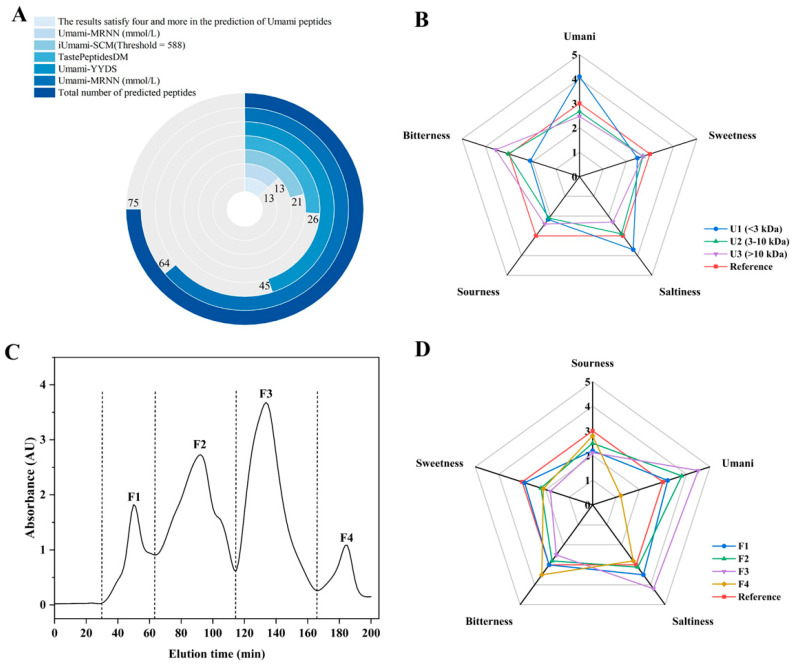
Identification and evaluation of potential umami peptides derived from protein hydrolysates. (**A**) Prediction results of 75 potential umami peptides by iUmami-SCM, Umami-MRNN, UMPred-FRL, Umami-YYDS, and TastePeptidesDM; (**B**) Sensory evaluation of various ultrafiltration fractions; (**C**) Gel filtration chromatogram of ultrafiltration fractions (<3 kDa); (**D**) Sensory evaluation of subfractions.

The umami peptide predictors were models developed using established taste profiles and amino acid profiles of flavor-presenting peptides, with the objective of forecasting umami properties and potential intensity of particular peptides [7,9,10,34]. The prospective umami peptides were identified on the basis of the predictive findings (Table S2). To enhance accuracy, these peptides were included in further research by being validated by four or more predictors. The results of the screening were shown in Figure 1A, in which 13 peptides exhibited satisfactory umami peptides.

Although in silico enzymatic hydrolysis and molecular docking offer efficient and cost-effective approaches for preliminary screening of umami taste peptides, these computational strategies are inherently limited by the simplifications embedded in their algorithms. The accuracy of docking results largely depends on the scoring functions employed, which may inadequately capture solvent effects, peptide flexibility, and conformational entropy [39]. Consequently, the predicted binding affinities may not fully reflect the actual enzymatic cleavage efficiency or biological activity observed under experimental conditions. Furthermore, the possibility of false-positive predictions cannot be excluded, particularly when the structural dynamics of peptide-enzyme interactions are oversimplified. To enhance the biological relevance and reliability of the computational predictions, a complementary peptide library was constructed based on the overlapping peptide fragments identified from actual enzymatic hydrolysis experiments. Integrating experimentally verified peptide sequences with virtual screening outcomes provides a more physiologically meaningful dataset and helps to minimize the overestimation of binding interactions. Furthermore, molecular dynamics simulations were performed to refine docking poses and reduce false-positive interactions, thereby enhancing the robustness of the virtual screening outcomes.

### 3.3. Taste Analysis and Separation Purification of Skipjack Tuna Protein Hydrolysate (SPHs)

The sensory characteristics for ultrafiltration fractions of SPHs were obtained as illustrated in Figure 1B, in which the U1 fraction exhibited pronounced umami and salty characteristics compared to U2 and U3, with milder sour and bitter notes. It has been ascribed that the pronounced saltiness may result from the interaction between salts and umami in the sample, with umami featuring an enhancing effect on saltiness [40]. It was notable that the U2 and U3 fractions exhibited weaker umami and bitter flavors, indicating that the primary flavor-active substances were enriched in the lower molecular weight fraction (<3 kDa). This finding conformed closely to the molecular weight distribution characteristics of umami-active peptides reported in the literature [31]. Therefore, the enriched U1 fraction was further separated using a dextran gel chromatography column, resulting in four fractions: F1–F4 (Figure 1C). It was observed that at equivalent concentrations, fraction F3 exhibited the strongest umami flavor, followed by F2, F1, and F4 (Figure 1D). Furthermore, fraction F3 demonstrated pronounced saltiness and the weakest bitterness. It was evident that minor molecules entering the gel pores during dextran gel separation experienced significant retention, leading to delayed elution. Consequently, umami intensity exhibited a pattern of increasing with molecular weight followed by a decrease, which was similar to the phenomenon observed in previous studies [41]. Therefore, F3 was gathered and harvested for peptide sequence identification.

### 3.4. Identification and Screening of Umami Peptides from SPH-F3 and Virtual Hydrolysis

A total of 3163 peptide sequences were obtained through sequence identification of SPH-F3 and evaluated on the basis of umami peptide characteristics, in terms of length, molecular weight, and iUmami-SCM predicted umami scores. Subsequently, the identified peptides from the SPH-F3 described above were matched against a virtual prediction library, which resulted in 10 common peptides being selected as umami peptide candidates, with the sequences, water solubility, and safety data presented in Table 1. The phenomenon of obtaining peptides with identical sequences validated the reliability of the virtual model screening approaches. However, there were still multi-source peptides detected in the actual SPH, while enzymatic digestion focused on bonito meat, which may be attributed to competitive hydrolysis of other proteins during the actual digestion process [42]. Therefore, the integration of the entire bonito proteome data is required in the future to enhance the predictive coverage.

### 3.5. Construction and Optimization of the T1R1/T1R3 Receptor

**Figure 2:**
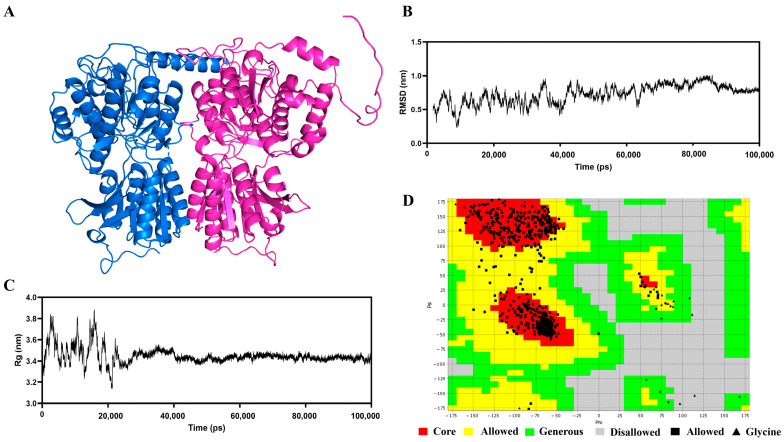
Results of the construction and optimization of the T1R1/T1R3 protein receptor. (**A**) Initial structure of T1R1/T1R3 receptor protein; (**B**) Rg values (nm) of the T1R1/T1R3 receptor protein by 100 ns MD simulation; (**C**) RMSD values (nm) of the T1R1/T1R3 receptor protein by 100 ns MD simulation; (**D**) Ramachandran plot of T1R1/T1R3 receptor protein after optimization.

Figure 2A illustrates the protein architectures of the T1R1 and T1R3 receptors predicted by AlphaFold2. The secondary framework of the T1R1/T1R3 receptor protein exhibited minor alterations subsequent to 100 nanoseconds of optimizing molecular dynamics simulations, as seen in Figure 2B,C, accompanied by a reduction in the range of Rg values. The fluctuations range diminished after 30 ns, indicating that the T1R1 and T1R3 receptor proteins were more compact upon binding. The RMSD readings exhibited a progressive stabilization after 70 ns [31]. The RMSD values stayed fairly consistent, signifying that the T1R1/T1R3 receptor protein achieved a firm structure. Figure 2D illustrates the Rasch diagram utilized for assessing the construction. The Rasch diagram concept evaluates the plausibility of amino acid locations according to Psi and Phi conformations [43]. The quantity of amino acid residues found within the favorable range of the Rasch plot exhibited a positive correlation with structural integrity, with amino acids situated between the yellow and green regions classified as borderline. Furthermore, all regions within the green area were considered acceptable, while the gray amino acids located outside the green region revealed that the amino acid positions lacked reasonable justification. This structure exhibited that 99.1% (>90%) of the observed residues were situated in the permission region (92.4% in the state of greatest favor region and 6.7% in the extra permission region), indicating that the structure was valid and further substantiating its reliability for molecular docking studies.

### 3.6. Molecular Docking Analysis of Umami Peptides Interacting with T1R1/T1R3

The Extracellular Venus flytrap domain (VFTD) is common to T1R1 and T1R3, both of which are classified as C-type G protein-coupled receptors (class C GPCRs). The principal umami receptor is T1R1/T1R3, which elicits an umami taste sensation upon the binding of the ligand to the receptor’s active site within the T1R1/T1R3 cavity [37,44]. Table 1 presents a summary of the affinity energy values for 10 peptides that have been docked with the T1R1/T1R3 receptor proteins. Prior research indicated a significant inverse association that exists between the intensity of umami taste and the affinity energy observed in molecular docking, revealing that umami peptides with lower affinity energy values exhibited a more pronounced umami flavor [4]. In this research, the umami peptides exhibiting an affinity energy of −8.0 kcal/mol were docked to the T1R1/T1R3 receptor; those with affinity energies lower than −8.0 kcal/mol were identified as promising candidates for umami peptides worthy of further investigation for their stable binding to umami receptors [43]. The binding energies were analyzed to assess five potential new umami peptides in relation to their interaction with the T1R1/T1R3 receptor protein. The GVGGHGAGG (GG-9) demonstrated the highest affinity of −9.2 kcal/mol among the promising umami peptides, followed by GVTGVG (GG-6), GGVAGCQGK (GK-9), MANR (MR-4), and SPAAK (SK-5).

### 3.7. Prediction of Umami Intensity

The umami strength of peptides is forecasted with BIOPEP-UWM. By quantifying the occurrence of umami-related amino acid segments, the umami intensity of prospective umami peptides may be anticipated and studied more effectively [45]. As shown in Table 2, the peptide fragments V, G, and VG concurrently exhibit three characteristics: umami, bitter, and sweet, which canincreaset the flavor complexity of peptides, while fragments containing the amino acid K mostly possess bitter, salty, and sour attributes. The peptide exhibiting the highest prevalence of umami amino acids was GG-9, with a frequency of 50%, comprising three umami amino acid fragments: V, G, VG, and VGG, as hydrophilic amino acids facilitate umami expression [45]. The GG-6 took second place in umami intensity, which contained one umami fragment, VG, while other umami peptide fragments of GK-9, MR-4, and SK-5 possessed no matches in the database of basic flavors. It has been demonstrated that active fragments and amino acid residues exert certain effects on the gustatory activity of peptides [42]. The predictions of BIOPEP-UWM for the umami peptides GG-9 (the strongest) and MR-4 (the weakest) were consistent in magnitude with those predicted for the binding energies in molecular docking.Previous researchh revealed that Asp, Glu, Phe, Ala, Gly, and Tyr have been identified as umami amino acids, while hydrophobic amino acids also constitute essential components of umami peptides [37]. Theabove-mentioned peptides above contain several residues commonly associated with umami and hydrophobic properties, including Gly, Ala, and Val, which provide structural flexibility and enable the formation of stable conformations for receptor binding. The database provided a certain reference and theoretical basis for studying flavor presentation properties. Apart from that, a descriptive flavor analysis and sensory assessment of the produced peptides should be used to confirm the flavor and taste properties of the synthesized peptides.

### 3.8. Intermolecular Interaction Force Analysis

**Figure 3:**
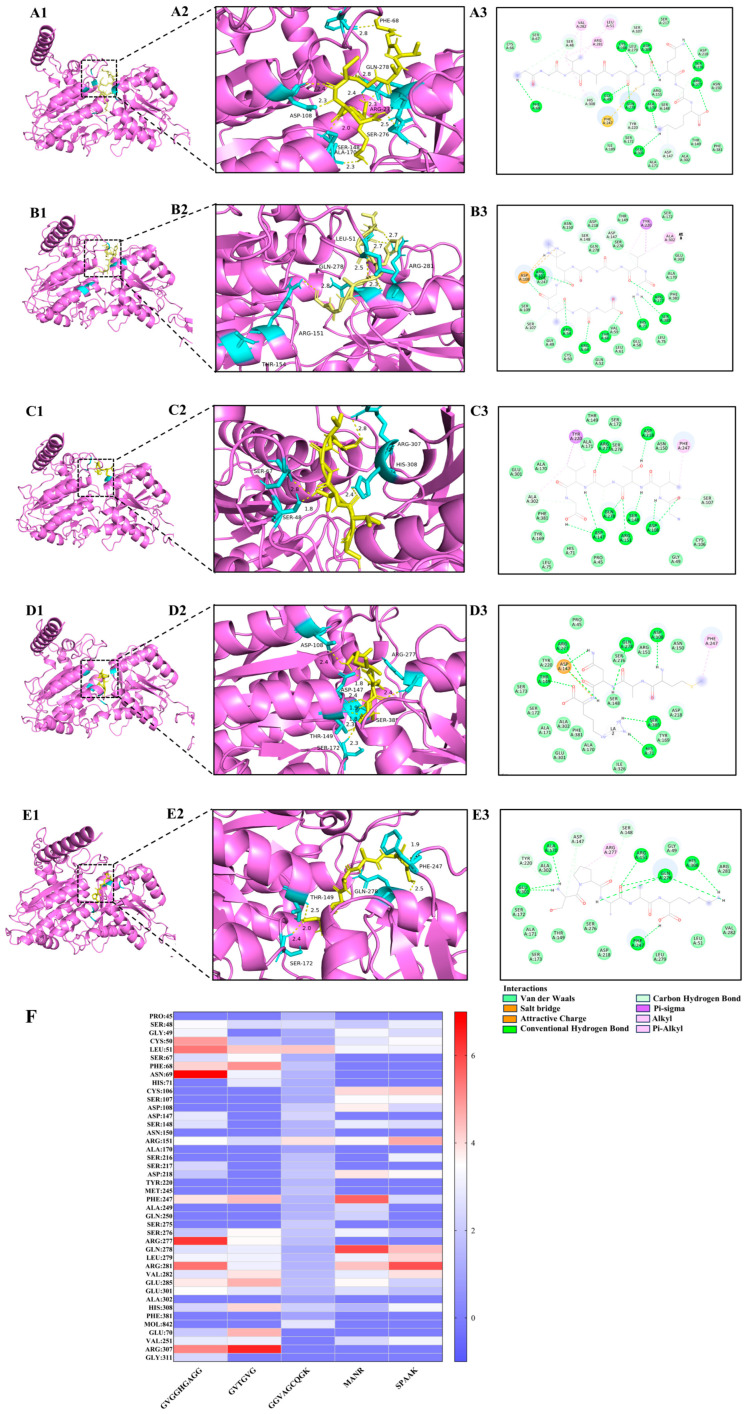
Results of molecular docking poses and the combined state of umami peptides. (**A1**–**A3**) GK-9, (**B1**–**B3**) GG-9, (**C1**–**C3**) GG-6, (**D1**–**D3**) MR-4, (**E1**–**E3**) SK-5 with the T1R1/T1R3 receptor protein. (**F**) Heat map results of hydrogen bonds between five potential umami peptides and amino acid residues of the T1R1/T1R3 receptor protein. The numbers 1–2, respectively, represent the binding site and intermolecular interaction force of 2D images of umami peptides and T1R1/T1R3 receptor protein.

Umami peptides predominantly engage with the T1R1/T1R3 receptor protein on the basis of hydrogen bonds, possibly complemented by van der Waals forces, electrostatics, and the presence of alkyl groups [46]. Short-range hydrogen bonds demonstrated robust binding capacity and created complexes with comparatively stable conformations. The quantity and arrangement of these hydrogen bonds that associate the T1R1/T1R3 receptor proteins with umami peptides were illustrated in Figure 3 and detailed in Table S3. The amino acid residues within the T1R1/T1R3 receptor protein generated 9, 7, 7, 6, and 6 hydrogen bonds with the umami peptides GG-9, GG-6, GK-9, MR-4, and SK-5, respectively. Meanwhile, the amino acid residues within the T1R1/T1R3 receptor exhibited 19, 21, 16, 16, and 15 van der Waals interactions with umami peptides GG-9, GG-6, GK-9, MR-4, and SK-5, resulting in the formation of 3, 1, 1, 1, 1, 1, and 1 alkyl groups. The findings on intermolecular interaction forces demonstrated that the umami peptides GG-9, GG-6, GK-9, MR-4, and SK-5 exhibited strong binding affinity for the T1R1/T1R3 receptor protein, involving hydrogen bonding, van der Waals forces, and alkyl contacts. Among these, GG-9 stood out with the highest binding affinity for the T1R1/T1R3 receptor, as measured by interaction force quantities. Furthermore, the hydrogen bond lengths connecting the T1R1 receptor protein with the five umami peptides presented in this work were predominantly under 3 Å, suggesting that the majority of the complexes generated were conformationally stable once more [31].

Molecular docking elucidated the primary active site, and Figure 3F revealed the crucial amino acid residues within the T1R1/T1R3 receptor protein that established hydrogen bonds upon exposure to five prospective umami peptides explored during our investigation [47]. These binding sites consist of the amino acid residues Ser48, Leu51, Arg151, Phe241, Arg281, Arg307, Clu285, and Glu301, with particular emphasis on Arg151 and Clu285, which may establish hydrogen bonds with each of the five peptides. Previous studies have demonstrated that Ser, Glu, Leu, and Arg are the primary amino acids in umami peptides screened from soy sauce [48], corn fermentation powder [24], and aqueous extract of the clam *M. meretrix* Linnaeus [49] that associate with the umami receptors T1R1/T1R3, performing a crucial role in umami synergy. These findings established the basis for the attachment of extra umami peptides to their receptors.

### 3.9. Molecular Dynamics (MD) Simulation

**Figure 4:**
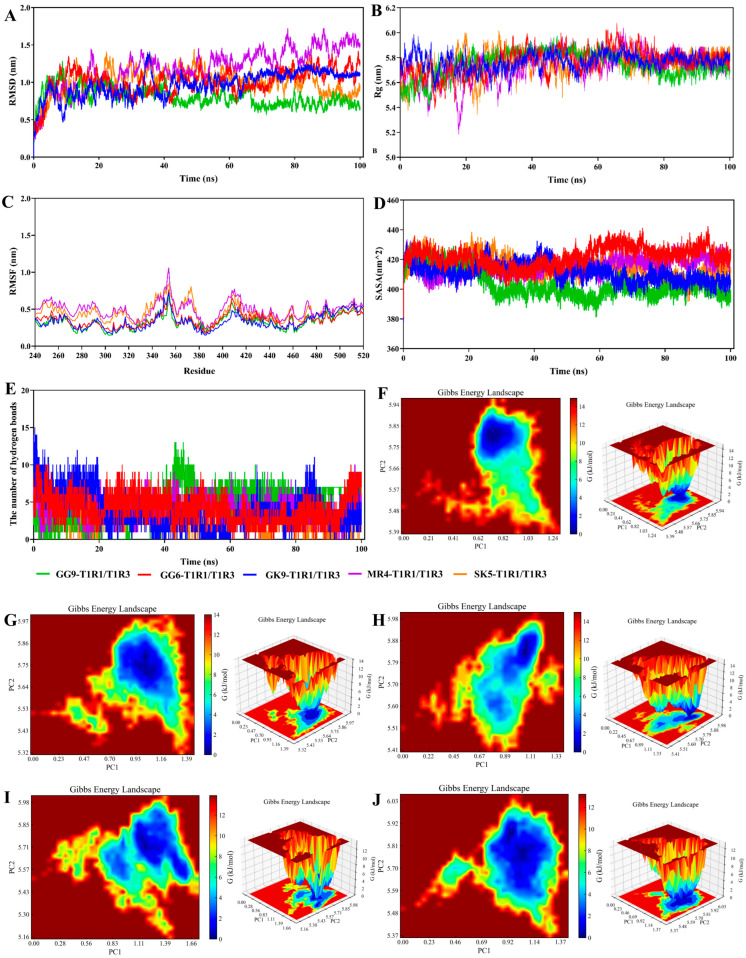
Molecular dynamics (MD) simulation analyses of the complexes between the umami peptide and the T1R1/T1R3 receptor. (**A**) RMSD; (**B**) Rg; (**C**) RMSF; (**D**) SASA; (**E**) The number of hydrogen bonds; (**F**) The free energy landscape map of GG-9; (**G**) The free energy landscape map of GG-6; (**H**) The free energy landscape map of GK-9; (**I**) The free energy landscape map of MR-4; (**J**) The free energy landscape map of SK-5.

During the binding process, the umami peptides were subjected to conformational shifts that could affect their interaction with the receptor. The examination of intermolecular contact forces relied on the structural characterization derived from molecular docking analysis, which solely accounts for the static binding of flexible ligands and stiff receptors, hence failing to adequately represent dynamic intermolecular interactions [50]. (MD simulations provided insight into the dynamic binding process of peptides to receptors. Extended kinetic simulations were capable of being used to more precisely forecast the binding instability of umami peptides to receptors. In the present investigation, the binding of protein to small molecules was projected by Gromacs MD simulation to assess T1R1/T1R3 receptor protein and umami peptides (GG-9, GG-6, GK-9, MR-4, and SK-5) interactions (Figure 4). The RMSD acted as a parameter for evaluating structural alterations in response to the protein. Figure 4A illustrates the binding stability of the T1R1/T1R3 receptor protein with each umami peptide (GG-9, GG-6, GK-9, MR-4, and SK-5) by the calculation of their RMSDs. The findings revealed that the RMSD values between the T1R1/T1R3 receptor protein and umami peptides were substantial in the initially occurring 20 ns of the simulation, then stabilized with time. The variations in the RMSD values resulted from the instability of the relative locations between the protein and peptides at the beginning of the MD simulation. Moreover, they led to the inadequacy of the system’s thermodynamic equilibrium [51]. Throughout the simulations, the RMSD values for GG-9-T1R1/T1R3, GG-6-T1R1/T1R3, GK-9-T1R1/T1R3, MR4-T1R1/T1R3, and SK5-T1R1/T1R3 stabilized within a narrow range (70–90 ns), suggesting that umami peptides associated with T1R1/T1R3 exhibited relative stability throughout the modeling procedures.

The variation in Rg values in the MD simulation provided a valuable measure for evaluating the entire compactness of a protein [52]. The gmx rotational software was utilized to compute the range of Rg values for each umami peptide (GG-9, GG-6, GK-9, MR-4, and SK-5) within the complicated system, including the T1R1/T1R3 receptor protein (Figure 4B). The experimental evidence confirmed that the Rg variation for the GG-9-T1R1/T1R3 complex remained minimal throughout the 100 ns MD simulation, fluctuating in the range of 5.75 and 5.85 nm. Conversely, the ranges of Rg values for the conjugates GG-6, GK-9, MR-4, and SK-5-T1R1/T1R3 exhibited a declining trend, attributed to the attachment of T1R1/T1R3 receptor proteins with peptides, which reduced the Rg value ranges compared to T1R1/T1R3 receptor proteins in isolation, suggesting that peptide binding renders the T1R1/T1R3 receptor proteins more compact.

The RMSF curve demonstrated the degree of variability of amino acid residues within protein occurring throughout the dynamical simulation, shedding light on the binding instability of the T1R1/T1R3 receptor for each peptide (Figure 4C) [47]. It was found that the RMSF curves for umami peptides GG-9, GG-6, GK-9, MR-4, SK-5, along with the T1R1/T1R3 receptor protein, fluctuated within a scope of 0.6 nm. The inclusion of GG-9, GG-6, GK-9, MR-4, and SK-5 exhibited an insignificant influence on the general structural stabilization of the T1R1/T1R3 receptor protein. Those particular amino acids displayed fluctuations close to 1.2 nm in the time range of 350–380 ns, which may be due to the fact that they were located at the edge of the T1R1/T1R3 receptor proteins, interacting with other elements in the complex, and thus generating variations in the simulation process.

SASA was employed to analyze the solvent-accessible surface area of a protein molecule, quantifying the exposed surface area of each atom and assessing their interactions and the stability of the protein structure [16]. For the present simulation, SASA was calculated for the umami peptides GG-9, GG-6, GK-9, MR-4, and SK-5 (Figure 4D). The findings indicated that the overall SASA of the complex constituted by the five umami peptides and the T1R1/T1R3 receptor protein significantly decreased after binding. The attachment of umami peptides resulted in a decrease in the surface area of protein, attributing to the interaction caused by umami peptides made with hydrophilic amino acid residues located within the T1R1/T1R3 lumen, which induced reduced hydrophilicity and the overall SASA [47]. The SASA values stabilized during the later simulation period (fluctuation range <5%), signifying that the complex reached a conformational equilibrium state where the solvent-accessible surface area at the binding interface showed no significant further changes. Notably, the GG-9-T1R1/T1R3 complex exhibited the greatest reduction in hydrophobic SASA owing to GG-9 binding to hydrophilic amino acid residues within the T1R1/T1R3 receptor protein cavity, constituting a stable complex that diminished hydrophilicity and total SASA. This implied that the GG-9-T1R1/T1R3 complex structure might possess higher thermal stability due to increased burial of hydrophobic interfaces, diminishing the risk of solvent-mediated denaturation.

The main mechanism that promotes umami peptides interacting with the T1R1/T1R3 receptor protein is hydrogen bonding, playing a vital role in maintaining protein structure. Figure 4E illustrates that every single umami peptide establishes multiple hydrogen bonds with the T1R1/T1R3 receptor protein, with binding stability potentially gauged by measuring the frequency of these interactions [50]. The data collected indicated that there was little change in the number of hydrogen bonds that were created when GG-9 interacted with the T1R1/T1R3 receptor protein in the presence of peptides GG-9, GG-6, GK-9, MR-4, and SK-5. Considering the dynamic nature of MD simulations, the association involving the T1R1/T1R3 protein receptor and umami peptides was observed to be stable, as fluctuations in the amount of hydrogen bonds created that linked the ligands and the protein [24]. Moreover, the 100 ns molecular dynamics simulation revealed many hydrogen bonds that connected the T1R1/T1R3 receptor protein and the umami peptides GG-6, GK-9, MR-4, and SK-5. This occurs owing to the generation of many hydrogen bonds involving the protein and tiny molecules throughout the simulation, mostly governed by hydrogen bonds between specific critical residues, primarily driven by connections between key residues in the protein and critical groups within the molecules.

Free Energy Landscape (FEL) reflected the interaction and energy distribution between molecules in a system and can better represent the interaction between molecules, conformation, stability, and other characteristics [53]. The peaks and low free energy areas of the free energy graph often signify energetically stable conformations, whereas robust and steady contacts can induce the development of nearly solitary and uniform energy clusters within the potential motivation landscape. Conversely, the free energy environment would show several shallow minima scattered across its surface if the interactions concerning the protein and the ligand were insufficient or prone to instability [54]. This simulation generates graphs based on RMSD and Rg. Figure 4F–J illustrate the presence of the virtually singular and smooth lowest energy cluster in the free energy conditions plots obtained for the GG-9-T1R1/T1R3, GG-6-T1R1/T1R3, and SK-5-T1R1/T1R3 composites, indicating a relatively stable conformation for these complexes. Furthermore, the free energy landscape maps revealed the presence of two low-energy regions within the GK-9-T1R1/T1R3 and MR-4-T1R1/T1R3 complexes. This suggested that the complex was poorly structured by the GK-9-T1R1/T1R3, MR-4-T1R1/T1R3, or that the system consisted of two or more conformations close to the energy-stabilized conformations, and these low-energy regions might correspond to molecules that reach the lowest energy through different interaction modes or conformations.

In summary, molecular interactions involving umami peptides with the T1R1/T1R3 receptor protein had been simulated utilizing Gromacs, with the results analyzed by several methods. The results verified that the contacts involving the umami peptides GG-9, GG-6, GK-9, MR-4, SK-5, and the T1R1/T1R3 receptor protein remained consistent over periods of 100 ns during simulations, illustrating that these five peptides possess the potential to function as umami peptides. Furthermore, the protein molecule experienced compaction when the umami peptide GG-9 bound to the T1R1/T1R3 receptor protein, contributing to a reduction in surface area. The interaction between GG-9 and T1R1/TIR3 exhibits a greater degree of stability with respect to GG-6, GK-9, MR-4, and SK-5, which formed numerous hydrogen bonds with the receptor protein, presenting umami characteristics and demonstrating the greatest potential to serve as a novel umami peptide. Consequently, subsequent studies were conducted to validate the biosynthesis of these five peptides.

### 3.10. Sensory Evaluation of Synthetic Peptides

**Figure 5:**
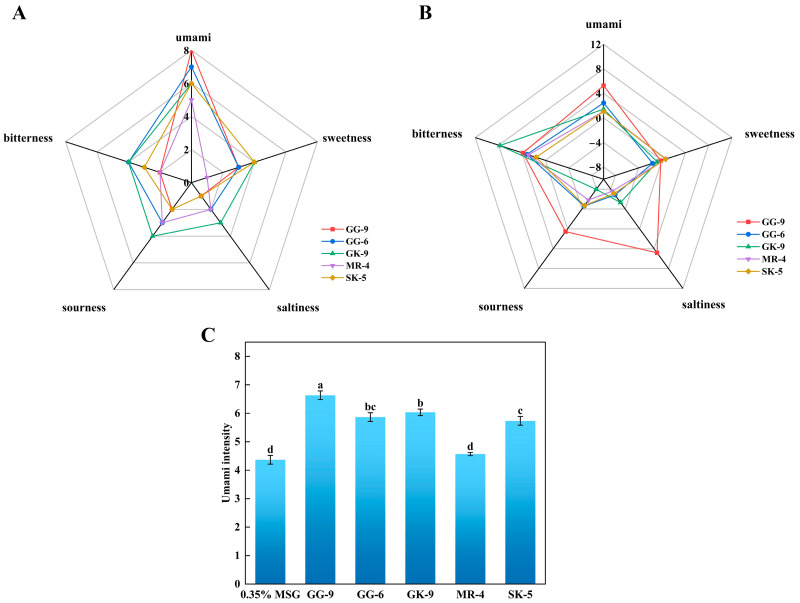
Taste characteristics of synthetic peptides by electronic tongue and sensory evaluation. (**A**) Taste profiles of sensory evaluation (1 mg/mL); (**B**) Taste profiles of electronic tongue (0.1 mg/mL); (**C**) umami-enhancing characteristics of the synthetic peptides. Different lowercase letters indicate significant differences (*p* < 0.05).

The three principal feelings of sour, bitter, and umami were incorporated in the sensational evaluation of the five umami peptides, which was shown in Figure 5A and Table 3. Out of all the umami peptides, GVGGHGAGG (GG-9) (7.3) had the highest umami peptide score (*p* < 0.05). This outcome aligned with the hypothesis that GG-9 bindings exhibited a greater stability to T1R1/T1R3 receptors compared to other umami peptides in molecular dynamics simulations. This result demonstrated the precision of the screening strategy employed in this work and its ability to forecast umami peptides of the same sequence length regarding umami taste strength. The sensory assessment panelists assessed the umami features of the synthetically manufactured peptides by analyzing the umami thresholds by TDA and providing sensory descriptions of the tastes encountered. The findings indicated that the thresholds for the synthetic peptides GG-9, GG-6, GK-9, MR-4, and SK-5 were 0.125 mg/mL, 0.25 mg/mL, 0.25 mg/mL, 0.75 mg/mL, and 0.5 mg/mL, respectively. The synthetic peptide GG-9 exhibited a markedly greater umami peptide taste intensity in sensory evaluations compared to GG-6, GK-9, MR-4, and SK-5, corroborating the predictive outcomes from molecular docking and kinetic simulations, hence affirming the reliability of this predictive methodology.

### 3.11. Electronic Tongue Evaluation

The electronic tongue assessment of five synthesized derived peptides with the two main flavors of umami and bitterness was illustrated in Figure 5B. Multiple investigations indicated that umami and bitterness were differentiated in the electronic tongue examination involving synthetic umami peptides, with hydrophobic amino acids likely being the primary contributors to bitterness. The electronic tongue analysis revealed that synthetic peptide GG-9 exhibited the most intense umami flavor, followed by GG-6, GK-9, MR-4, and SK-5, corresponding to the results of sensory evaluation, binding energy analysis in molecular docking, and kinetic simulation. Additionally, all synthetic peptides exhibited various degrees of bitterness, with the potential association being hydrophobic amino acids within their structures [55]. Simultaneously, GG-9 displayed salty, sour, and slightly bitter tastes. Studies implicated the presence of acidic amino acid residues and acetate ions during synthesis as potential contributors to the perceived sourness of synthetic peptides [42,43].

With a view to further evaluating the umami-enhancing effects of the identified peptides, the five peptide compounds were incorporated into a 0.35% MSG medium to assess their capacity for enhancing umami perception. Figure 5C depicted that the incorporation of 1 mg/mL of GG-9, GG-6, and MR-4 markedly increased the umami strength of the 0.35% MSG medium (*p* < 0.05), maybe due to the synergistic effects among these three umami peptides in conjunction with MSG. The GK-9 and SK-5 did not exhibit significant umami-enhancing effects in this investigation (*p* > 0.05), attributable to their pronounced sour and bitter flavors. Although MSG is known to mask bitterness and sourness through receptor-mediated synergistic interactions, in this case, the intrinsic sour and bitter characteristics of GK-9 and SK-5 may have outweighed the potential masking effect, preventing a notable increase in umami perception [56]. Furthermore, GG-9 demonstrated the most significant increase in umami peptides, followed by GG-6 and MR-4. It has been theorized that the presence of MSG induced synergistic umami enhancement by facilitating the conjugation of umami peptides with receptor proteins [57]. These findings aligned with previously documented data indicating that umami peptides exhibit high umami intensity and substantial taste enhancement in a 0.35% monosodium glutamate solution [16]. The results above revealed that the five potential umami peptides identified as a result of screening possess umami flavor but also effectively contribute to enhancing their intensity, rendering the solution with a more vigorous flavor profile.

### 3.12. Peptide Activity Prediction and Molecular Docking Analysis

To explore the potential biological activity of the above-mentioned umami peptides, functional activity predictions for five umami peptides performed utilizing BIOPEP-UWM revealed that portions exhibit antioxidant properties, as shown in Table 4. The analysis of active amino acid fragments in peptides revealed that GV (5), VG (3), GG (3), AG (1), GH (1), GK (1), HG (1), QG (1), TG (1), GHG (1), and AA (1) were identified as active fragments frequently associated with antioxidant activity and contained hydrophobic amino acids. In particular, the amino acid fragments corresponding to antioxidant activity in GG-9, GG-6, and GK-9 exhibited the highest frequency of occurrence, which may possess elevated antioxidant activity. With a view to further exploring antioxidant activity, five selected potential umami peptides were subjected to docking simulations with the active cavity of the antioxidant receptor protein Keap1. With the docking energy directly reflecting the stability of the receptor-ligand complex, a binding energy below −7 kcal/mol demonstrated an excellent binding affinity between the receptor and ligand, along with an elevated potential activity [58]. Specifically, the binding energies exhibited by peptides GG-9, GG-6, and GK-9 with Keap1 were all below -7 kcal/mol, implying stable peptide-Keap1 interactions and confirming their potential antioxidant activity (Table 4).

**Figure 6:**
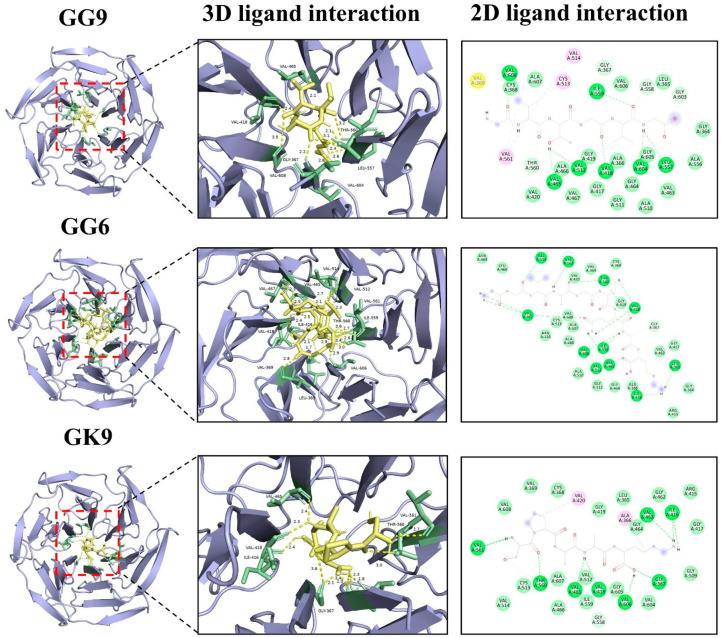
Molecular docking results of umami peptides (GG-9, GG-6, and GK-9) and Keap1 receptor protein.

The mechanism of molecular interactions between peptides and the Keap1 protein was analyzed, as depicted in Figure 6. The findings revealed that multiple hydrogen bonds form stable interactions between peptides and the Keap1 receptor protein, which was one of the principal forms of intermolecular interactions critical for maintaining protein structure and function. The peptides GG-9, GG-6, and GK-9 developed 7–9 hydrogen bonds with amino acid residues of the Keap1 receptor protein. The bond distances range from 1.7 to 3.6 Å., with the average bond distances to the receptor protein consistently below 3 Å, implying that the peptides bind stably to the receptor protein through hydrogen bond interactions [59]. Previous studies have confirmed that the potential for intermolecular hydrogen bonding formulation is a principal driver of peptide biological activity [60]. Furthermore, certain amino acid residues in hydrophobic regions (e.g., VAL608, ILE559, THR560, LEU557, GLY367) were observed to interact with the protein receptor via hydrophobic interactions, which may directly participate in hydrogen bonding, hydrophobic interactions, or electrostatic interactions [24]. These findings coincided with previous studies reporting that VAL [61], ILE [62], THR [63], and LEU [17] are the critical residues for the binding of the antioxidant peptide to the protein receptor, providing guidance for their interaction. The identified peptides, GVGGHGAGG, GVTGVG, and GGVAGCQGK, were predicted to interact with the Keap1 receptor protein, indicating a potential mechanism for activating the Nrf2-mediated antioxidant pathway. The presence of residues such as histidine and cysteine contributes to electron-donating and metal ion-chelating abilities, which are crucial for free radical scavenging and redox regulation. Hydrophobic and small residues (Gly, Ala, Val) may further stabilize the peptide-Keap1 complex by facilitating van der Waals and hydrogen-bond interactions within the Keap1 binding pocket. These characteristics are consistent with the findings of Rzhepakovsky et al. [64], who discovered that chicken embryo peptides exert biological functions through hydrophobic and sulfur-containing residues. It was observed that peptide size, hydrophobicity, and the presence of aromatic or charged residues are conserved determinants of antioxidant potential across animal and plant protein sources. These findings align with previous reports from barley distillers’ grain [58], bovine hemoglobin peptides [15], and chicken embryo studies [64], highlighting convergent structure-activity relationships. Therefore, this implied that peptides GG-9, GG-6, and GK-9 possessed potential antioxidant activity in addition to umami taste, raising the potential for developing functional umami peptides. In the investigation by Hao et al. [65], functional umami peptides with remarkable antioxidant activity and angiotensin I-converting enzyme inhibitory activity were identified on the basis of HPLC-MS/MS. The dual functionality of these peptides, combining sensory (umami-enhancing) and biological activities, suggests their potential use as natural flavor enhancers with added health benefits. Such multifunctional peptides could contribute to the development of next-generation functional foods that deliver both improved palatability and oxidative stress mitigation.

## 4. Conclusions

This work identified and analyzed five putative umami peptides (GVGGHGAGG, GVTGVG, GGVAGCQGK, MANR, and SPAAK) derived from skipjack tuna protein by a unique methodology that integrated molecular docking and molecular dynamics modeling. In molecular docking and molecular dynamics simulations, these five umami peptides demonstrated a consistent affinity (−9.2 to −8 kcal/mol) for the T1R1/T1R3 receptor protein through various intermolecular interactions, including hydrogen bonds, van der Waals forces, and alkyl associations. The highest binding stability was observed between GVGGHGAGG and T1R1/T1R3 proteins, with Arg151 and Clu285 identified as critical amino acid residues establishing the binding site. Sensory investigations and electronic tongue analysis confirmed the umami characteristics of these five proposed unique umami peptides (thresholds: 0.125–0.75 mg/mL), with GVGGHGAGG, GVTGVG, and MANR markedly increasing the umami strength of the 0.35% MSG medium (*p* < 0.05). Subsequent bioinformatics prediction and computational calculations revealed that umami peptides GVGGHGAGG, GVTGVG, and GGVAGCQGK interacted with the antioxidant receptor Keap1 through hydrogen bonds and hydrophobic interactions (energies < −7 kcal/mol, interactions with VAL608/ILE559/THR560), signaling their potential antioxidant activity. Nevertheless, this investigation suffered from certain limitations, including a lack of in vitro and in vivo verification of antioxidant efficacy and bioavailability. Future research should prioritize cellular assays (ROS scavenging and Nrf2 activation) and animal studies for bioactivity confirmation, simulated digestion and Caco-2 permeability studies for bioavailability assessment, and application trials in low-sodium food matrices. This work proposed that novel umami peptides can be subject to efficient and rapid evaluation based on computational analysis and that the prediction of biological activity and molecular simulation facilitates the development of umami peptide bioactivity, supporting their scalable application in the flavor enhancer and functional ingredient industries.

## Figures and Tables

**Table 1:** Characteristics and affinity energy of potential umami peptides identified based on hydrolysis and virtual screening.

Peptide	Abbreviation	Solubility	Toxicity	iUmami-SCM	Affinity Energy (kcal/mol)
GVGGHGVGG	GG-9	Good	Non-Toxin	Score = 648.27, Umami	−9.2
GVTGVG	GG-6	Good	Non-Toxin	Score = 590.36, Umami	−8.5
GGAGVP	GP-6	Good	Non-Toxin	Score = 668.0, Umami	−7.5
GA	GA-2	Good	Non-Toxin	Score = 617.35, Umami	−6.6
GVGG	GG-4	Good	Non-Toxin	Score = 595.78, Umami	−7.5
GGVAGCQGK	GK-9	Good	Non-Toxin	Score = 620.05, Umami	−8.4
SPAAK	SK-5	Good	Non-Toxin	Score = 599.76, Umami	−8
PGY	PY-3	Good	Non-Toxin	Score = 588.09, Umami	−7.6
MANR	MR-4	Good	Non-Toxin	Score = 589.65, Umami	−8
GVAT	GT-4	Good	Non-Toxin	Score = 590.54, Umami	−7.5

**Table 2:** Results of amino acid fragments corresponding to taste for predicting flavor presentation properties.

Sequences	Amino Acid Fragments Corresponding to Taste					Frequency of Occurrence of Umami
	Umami	Sweet	Bitter	Salty	Sour	
GG-9	VG, VGG	V, G	V, GV, VG	/	/	50%
GG-6	VG	V, G	V, GV, VG	/	/	33%
GK-9	/	K, V, G, A	V, GV, GGV, K, VA	K	K	0%
MR-4	/	A	/	/	/	0%
SK-5	/	K, AA, P, A	P, K, PA	K	K	0%

Note: / represents amino acid fragments not found in the corresponding flavors in the database for peptide inclusion.

**Table 3:** Taste description of synthetic peptides and their thresholds in water.

Sequence	Threshold Value (mg/mL)	Umami Scores
GG-9	0.125	7.3
GG-6	0.25	7
GK-9	0.25	6.2
MR-4	0.75	5.3
SK-5	0.5	6.4

**Table 4:** Results of amino acid fragments corresponding to taste for predicting antioxidant presentation properties and the affinity energy of peptides to the Keap1 receptor protein.

Sequence	Amino Acid Fragments	Frequency of Occurrence	Affinity Energy (kcal/mol)
GG-9	GV, VG, GH, GG, HG, GHG	0.67	−7.5
GG-6	GV, VG, TG	0.5	−7.3
GK-9	GG, GV, AG, QG, GK	0.45	−8.1
MR-4	/	0	−6.8
SK-5	AA	0.2	−6.5

## Data Availability

The original contributions presented in this study are included in the article/Supplementary Materials. Further inquiries can be directed to the corresponding authors.

## Supplementary Materials

The following supporting information can be downloaded at https://www.mdpi.com/article/10.3390/foods14213777/s1. Table S1: Results of solubility, toxicity, and peptide mass; Table S2: Prediction of reported umami peptides by iUmami-SCM, Umami-MRNN, UMPred-FRL, Umami-YYDS and TastePeptides DM. Table S3: Results of hydrogen bonding and van der Waals forces between five umami peptides and T1R1/T1R3 receptor protein by molecular docking.
