# Supplementary material for: The Screening Strategy and Activity Investigation of Skipjack Tuna (Katsuwonus pelamis) Umami Peptides Based on Computer Simulation Prediction and Experimental Hydrolysis
Source: Foods. 2025 Nov 4;14(21):3777. doi: 10.3390/foods14213777 (PMC12607353; doi:10.3390/foods14213777)
Supplement: Supplementary file 1 [file foods-14-03777-s001.zip › foods-3930539-supplementary.pdf]

The screening strategy and activity investigation of Skipjack tuna (*Katsuwonus pelamis*) umami peptides based on computer simulation prediction and experimental hydrolysis

Qiufeng Song[a, c#], Panpan Wang[a#], Huawei Wu[d], Weiliang Guan[b, *], Luyun Cai[a, *]

[a] *Ningbo Global Innovation Center, Zhejiang University, Ningbo 315100, China*

[b] *College of Light Industry and Food Engineering, Guangxi University, Nanning 530004, China*

c Engineering Research Center of Bio-Process, Ministry of Education, School of Food and Biological Engineering, Hefei University of Technology, Hefei 230009, China

[d] *Ningbo Today Food Company, Limited, Ningbo 315100, China*

*Corresponding authors: Weiliang Guan, E-mail address: wlguan@gxu.edu.cn;

Luyun Cai, E-mail address: cailuyun@zju.edu.cn

Table S1. Results of solubility, toxicity, and peptide mass.

Number	Peptide sequence	Estimated solubility	Toxicity	Peptide mass
1	MANR	Good	Non-required	490.578
2	NIL	Good	Non-required	358.438
3	CG	Poor	Non-required	178.22
4	IA	Poor	Non-required	202.27
5	IQPSL	Poor	Non-required	556.73
6	KPAK	Good	Non-required	442.6
7	AAGGY	Poor	Non-required	437.51
8	GAGAR	Good	Non-required	430.52
9	GVGAGG	Poor	Non-required	416.52
10	GVGG	Poor	Non-required	288.36
11	GN	Good	Non-required	189.19
12	GVGGHGAGG	Poor	Non-required	667.82
13	GVGGY	Poor	Non-required	451.55
14	GGY	Poor	Non-required	295.33
15	GGAGG	Poor	Non-required	317.37
16	PGAGQK	Good	Non-required	556.7
17	AAK	Good	Non-required	288.37
18	GAGAP	Poor	Non-required	371.45
19	PGGGPVL	Poor	Non-required	595.8
20	PQTGL	Poor	Non-required	514.65
21	VPGVGVPGL	Poor	Non-required	794.1
22	QGGMVSGK	Good	Non-required	763
23	GGR	Good	Non-required	288.34
24	GVL	Poor	Non-required	287.4
25	PGVATGTG	Poor	Non-required	658.82
26	LTPK	Good	Non-required	457.62
27	GY	Poor	Non-required	238.26

28	GGQMQPGV	Poor	Non-required	773
29	HGYP	Poor	Non-required	472.55
30	LK	Good	Non-required	259.37
31	SPK	Good	Non-required	330.41
32	VQGAY	Poor	Non-required	536.65
33	GAKPGGGK	Good	Non-required	670.88
34	PY	Poor	Non-required	278.32
35	GG	Poor	Non-required	132.14
36	GAGGAGL	Poor	Non-required	501.64
37	PGGK	Good	Non-required	357.46
38	GL	Poor	Non-required	188.25
39	LYPGAGY	Poor	Non-required	739.92
40	GAAAK	Good	Non-required	416.53
41	LAVPGGGL	Poor	Non-required	682.94
42	SPAAK	Good	Non-required	472.59
43	GVGAGGVGG	Poor	Non-required	629.81
44	GA	Poor	Non-required	146.16
45	GGHAGL	Poor	Non-required	510.64
46	AGAAAAK	Poor	Non-required	558.71
47	GAGGR	Good	Non-required	416.5
48	PGY	Poor	Non-required	335.39
49	GS	Good	Non-required	162.16
50	AAGAKPPK	Good	Non-required	738.98
51	GVPGAG	Poor	Non-required	456.58
52	GPY	Poor	Non-required	335.39
53	GVKP	Good	Non-required	399.54
54	GVPGGVGAG	Poor	Non-required	669.87
55	GAGAG	Poor	Non-required	331.39
56	GVTGVG	Poor	Non-required	488.63
57	LKPGK	Good	Non-required	541.76

58	PPKPIL	Good	Non-required	663.94
59	PASPY	Poor	Non-required	533.63
60	PGAKP	Good	Non-required	468.61
61	LKPPGGIP	Good	Non-required	778.08
62	LAAGGG	Poor	Non-required	444.57
63	TYPGAAG	Poor	Non-required	635.76
64	GGAGAK	Good	Non-required	459.58
65	APKPGY	Good	Non-required	631.8
66	GVGGG	Poor	Non-required	345.43
67	GGGYPQPY	Poor	Non-required	838
68	YPGGY	Poor	Non-required	555.65
69	VPAP	Poor	Non-required	382.5
70	LTPQQAK	Good	Non-required	785.01
71	GP	Poor	Non-required	172.2
72	LQG	Poor	Non-required	316.4
73	GGAGGVPR	Good	Non-required	669.85
74	GGVAGCQGK	Good	Non-required	776.01
75	CGR	Good	Non-required	334.42

Table S2. Prediction of reported umami peptides by iUmami-SCM、Umami-MRNN、UMPred-FRL、Umami-YYDS and TastePeptides DM

	Prediction result					
Peptide	Peptide size	iUmami-SCM	iUmami-MRNN	UMPred-FRL	Umami -YYDS	TastePeptidesDM
MANR	4	Score = 621.33, Umami	umami, predicted threshold: 31.262619mmol/L	Umami	Umami	Umami
NIL	3	Score = 592.0, Umami	non- umami	non-Umami	non-Umami	no-Umami
CG	2	Score = 604.0, Umami	umami, predicted threshold: 23.990171mmol/L	non-Umami	Umami	no-Umami
IA	2	Score = 468.0, non-Umami	non-umami	non-Umami	non-Umami	no-Umami
IQPSL	5	Score = 485.0, non-Umami	umami, predicted threshold: 28.924078mmol/L	non-Umami	Umami	Umami
KPAK	4	Score = 598.33, Umami	umami, predicted threshold: 38.633904mmol/L	non-Umami	non-Umami	no-Umami
AAGGY	5	Score = 516.75, non-Umami	umami, predicted threshold: 14.673149mmol/L	non-Umami	Umami	no-Umami
GAGAR	5	Score = 617.0, Umami	umami, predicted threshold: 8.1645mmol/L	Umami	Umami	Umami
GVGAGG	6	Score = 549.4, non-Umami	umami, predicted threshold: 29.563604mmol/L	non-Umami	Umami	Umami
GVGG	4	Score = 505.67, non-Umami	non-umami	Umami	non-Umami	no-Umami
GN	2	Score = 595.0, Umami	umami, predicted threshold: 24.66064mmol/L	Umami	Umami	Umami
GVGGHGAGG	9	Score = 527.75, non-Umami	umami, predicted threshold: 12.384403mmol/L	non-Umami	non-Umami	Umami
GVGGY	5	Score = 520.25, non-Umami	umami, predicted threshold: 33.44271mmol/L	non-Umami	Umami	no-Umami
GGY	3	Score = 437.5, non-Umami	umami, predicted threshold: 23.142166mmol/L	Umami	non-Umami	no-Umami
GGAGG	5	Score = 463.0, non-Umami	umami, predicted threshold: 22.627396mmol/L	non-Umami	Umami	no-Umami
PGAGQK	6	Score = 486.0, non-Umami	umami, predicted threshold: 10.988665mmol/L	non-Umami	Umami	Umami
AAK	3	Score = 615.5, Umami	umami, predicted threshold: 15.659719mmol/L	non-Umami	non-Umami	no-Umami
GAGAP	5	Score = 607.0, Umami	umami, predicted threshold: 10.092195mmol/L	non-Umami	non-Umami	Umami

PGGGPVL	7	Score = 341.33, non-Umami	non-umami	non-Umami	Umami	no-Umami
PQTGL	5	Score = 552.75, non-Umami	non-umami	non-Umami	Umami	Umami
VPGVGVPGL	9	Score = 502.62, non-Umami	umami, predicted threshold: 37.37888mmol/L	non-Umami	Umami	Umami
QGGMVSGK	7	Score = 547.14, non-Umami	umami, predicted threshold: 6.7811527mmol/L	Umami	Umami	Umami
GGR	3	Score = 421.5, non-Umami	umami, predicted threshold: 34.12211mmol/L	non-Umami	non-Umami	no-Umami
GVL	3	Score = 540.0, non-Umami	non-umami	non-Umami	Umami	no-Umami
PGVATGTG	8	Score = 516.29, non-Umami	umami, predicted threshold: 6.545717mmol/L	non-Umami	Umami	Umami
LTPK	4	Score = 569.67, non-Umami	umami, predicted threshold: 32.083874mmol/L	non-Umami	Umami	no-Umami
GY	2	Score = 564.0, non-Umami	umami, predicted threshold: 27.872505mmol/L	non-Umami	non-Umami	no-Umami
GGQMQPGV	8	Score = 483.71, non-Umami	umami, predicted threshold: 29.788387mmol/L	non-Umami	Umami	Umami
HGYP	4	Score = 527.33, non-Umami	umami, predicted threshold: 38.347496mmol/L	non-Umami	non-Umami	no-Umami
LK	2	Score = 596.0, Umami	umami, predicted threshold: 31.669472mmol/L	non-Umami	non-Umami	no-Umami
SPK	3	Score = 552.5, non-Umami	umami, predicted threshold: 35.163517mmol/L	non-Umami	non-Umami	no-Umami
VQGAY	5	Score = 594.25, Umami	umami, predicted threshold: 17.071417mmol/L	non-Umami	Umami	Umami
GAKPGGGK	8	Score = 486.43, non-Umami	umami, predicted threshold: 1.8924502mmol/L	non-Umami	Umami	Umami
PY	2	Score = 732.0, Umami	umami, predicted threshold: 22.665756mmol/L	non-Umami	non-Umami	no-Umami
GG	2	Score = 311.0, non-Umami	umami, predicted threshold: 37.460705mmol/L	non-Umami	Umami	no-Umami
GAGGAGL	7	Score = 547.83, non-Umami	umami, predicted threshold: 29.24352mmol/L	non-Umami	Umami	Umami
PGGK	4	Score = 415.0, non-Umami	umami, predicted threshold: 38.168915mmol/L	non-Umami	non-Umami	no-Umami
GL	2	Score = 516.0, non-Umami	non-umami	non-Umami	non-Umami	no-Umami
LYPGAGY	7	Score = 519.67, non-Umami	umami, predicted threshold: 35.657608mmol/L	non-Umami	Umami	no-Umami

GAAAK	5	Score = 619.25, Umami	umami, predicted threshold: 7.3187633mmol/L	non-Umami	Umami	no-Umami
LAVPGGGL	8	Score = 443.29, non-Umami	umami, predicted threshold: 21.54608mmol/L	non-Umami	Umami	no-Umami
SPAAK	5	Score = 601.75, Umami	umami, predicted threshold: 5.1776614mmol/L	Umami	Umami	no-Umami
GVGAGGVGG	9	Score = 533.0, non-Umami	umami, predicted threshold: 13.724253mmol/L	non-Umami	Umami	Umami
GA	2	Score = 642.0, Umami	umami, predicted threshold: 24.99813mmol/L	Umami	Umami	no-Umami
GGHAGL	6	Score = 515.8, non-Umami	umami, predicted threshold: 24.835184mmol/L	non-Umami	Umami	no-Umami
AGAAAAK	7	Score = 611.5, Umami	umami, predicted threshold: 2.235262mmol/L	non-Umami	Umami	Umami
GAGGR	5	Score = 518.25, non-Umami	umami, predicted threshold: 8.136755mmol/L	non-Umami	Umami	Umami
PGY	3	Score = 443.5, non-Umami	umami, predicted threshold: 33.910355mmol/L	non-Umami	non-Umami	no-Umami
GS	2	Score = 696.0, Umami	umami, predicted threshold: 22.4026mmol/L	Umami	Umami	no-Umami
AAGAKPPK	8	Score = 542.86, non-Umami	umami, predicted threshold: 19.52251mmol/L	non-Umami	Umami	no-Umami
GVPGAG	6	Score = 530.0, non-Umami	umami, predicted threshold: 18.3236mmol/L	non-Umami	Umami	no-Umami
GPY	3	Score = 423.0, non-Umami	umami, predicted threshold: 19.561304mmol/L	non-Umami	non-Umami	no-Umami
GVKP	4	Score = 567.67, non-Umami	umami, predicted threshold: 35.7864mmol/L	non-Umami	non-Umami	no-Umami
GVPGGVGAG	9	Score = 520.88, non-Umami	umami, predicted threshold: 10.004459mmol/L	non-Umami	non-Umami	Umami
GAGAG	5	Score = 615.0, Umami	umami, predicted threshold: 19.340845mmol/L	non-Umami	Umami	no-Umami
GVTGVG	6	Score = 595.8, Umami	umami, predicted threshold: 8.328091mmol/L	non-Umami	Umami	Umami
LKPGK	5	Score = 527.5, non-Umami	umami, predicted threshold: 32.87623mmol/L	non-Umami	Umami	no-Umami
PPKPIL	6	Score = 461.4, non-Umami	non-umami	non-Umami	non-Umami	no-Umami
PASPY	5	Score = 628.0, Umami	umami, predicted threshold: 26.931355mmol/L	non-Umami	non-Umami	no-Umami

PGAKP	5	Score = 543.0, non-Umami	umami, predicted threshold: 17.20478mmol/L	non-Umami	Umami	no-Umami
LKPPGGIP	8	Score = 375.14, non-Umami	umami, predicted threshold: 39.43772mmol/L	non-Umami	non-Umami	no-Umami
LAAGGG	6	Score = 462.4, non-Umami	umami, predicted threshold: 39.530968mmol/L	non-Umami	Umami	no-Umami
TYPGAAG	7	Score = 529.17, non-Umami	umami, predicted threshold: 26.257397mmol/L	non-Umami	Umami	Umami
GGAGAK	6	Score = 562.0, non-Umami	umami, predicted threshold: 4.641033mmol/L	Umami	Umami	no-Umami
APKPGY	6	Score = 508.0, non-Umami	umami, predicted threshold: 7.8045664mmol/L	non-Umami	Umami	no-Umami
GVGGG	5	Score = 457.0, non-Umami	umami, predicted threshold: 37.853584mmol/L	non-Umami	Umami	no-Umami
GGGYPQPY	8	Score = 496.29, non-Umami	umami, predicted threshold: 23.75198mmol/L	non-Umami	non-Umami	Umami
YPGGY	5	Score = 408.5, non-Umami	non-umami	non-Umami	non-Umami	no-Umami
VPAP	4	Score = 566.67, non-Umami	umami, predicted threshold: 29.389395mmol/L	Umami	non-Umami	no-Umami
LTPQQAK	7	Score = 609.67, Umami	umami, predicted threshold: 17.940718mmol/L	non-Umami	Umami	Umami
GP	2	Score = 114.0, non-Umami	non-umami	non-Umami	non-Umami	no-Umami
LQG	3	Score = 595.5, Umami	non-umami	non-Umami	non-Umami	no-Umami
GGAGGVPR	8	Score = 505.29, non-Umami	umami, predicted threshold: 4.9851933mmol/L	non-Umami	non-Umami	Umami
GGVAGCQGK	9	Score = 556.38, non-Umami	umami, predicted threshold: 0.5970904mmol/L	Umami	Umami	Umami
CGR	3	Score = 568.0, non-Umami	umami, predicted threshold: 29.915442mmol/L	Umami	non-Umami	no-Umami

Table S3. Results of hydrogen bonding and van der Waals forces between five umami peptides and T1R1/T1R3 receptor protein by molecular docking.

Umami peptide	Hydrogen bonds			Van der Waals forces	
	number	amino acid residues	average distance (Å)	number	amino acid residues
GGVAGCQGK	9	PHE68,GLY49,GLN278,ALA170,GLU301,ARG277,SER276,ASP108,CYS106	2.42	19	CYS66,SER67,SER48,SER107,LEU279,SER217,ASP218,ASN150,PHE381,THR149,ALA302,ALA171,SER172,ILE189,TYR220,SER148,ARG151,HIS308,ASP157
GVGGHGAGG	7	ARG151,ARG64, ARG54,THR60,HIS71,SER385,ARG277	2.52	21	PHE247,SER109,SER107,GLY49,CYS50,GLN52,LEU61,VAL59,GLU58,LEU75,PHE381,ALA170,GLU301,SER172,THR149,SER276,ASP147,ASP218,SER148,ASN150，GLN278
GVTGVG	7	ASP147,GLN278,SER148,ARG151,ASP108,ASP218,ARG277	2.27	16	GLU301,ALA170,ALA302,PHE381,TYR169,LEU75,HIS71,PRO45,GLY49,CYS106,SER107,ASN150,SER276,SER172,ALA171,THR149
MANR	6	THR149,ARG277,GLN278,ASP108,SER385,HIS71	2.12	16	PRO45,TYR220,SER173,SER172,ALA171,ALA302,PHE381,GLU301,ALA170,ILE326,TYR169,ASP218,SER148,ASN150,ARG151,SER276
SPAAK	6	GLU301,ALA170,ARG151,GLN278,HIS308,PHHE247	2.26	15	ASP147,TYR220,ALA302,SER172,ALA171,THR149,SER173,SER276,ASP218,LEU279,LEU51,VAL282,ARG281,GLY49,SER148
